# First multi-bend achromat lattice consideration

**DOI:** 10.1107/S160057751401193X

**Published:** 2014-08-27

**Authors:** Dieter Einfeld, Mark Plesko, Joachim Schaper

**Affiliations:** a MAX IV Laboratory, Lund University, PO Box 118, Lund SE-221 00, Sweden; b COSYLAB, Teslova ulica 30, Ljubljana SI-1000, Slovakia; c HAWK University of Applied Sciences and Arts, Hohnsen 4, D-31134 Hildesheim, Germany

**Keywords:** synchrotron light source, beam optics, lattice, DLSR, MAX IV

## Abstract

By the beginning of 1990, three third-generation synchrotron light sources had been successfully commissioned in Grenoble, Berkeley and Trieste (ESRF, ALS and ELETTRA). Each of these new machines reached their target specifications without any significant problems. In parallel, already at that time discussions were underway regarding the next generation, the ‘diffraction-limited light source (DLSR)’, which featured sub-nm rad electron beam emittance, photon beam brilliance exceeding 10^22^ and the potential to emit coherent radiation. Also, at about that time, a first design for a 3 GeV DLSR was developed, based on a modified multiple-bend achromat (MBA) design leading to a lattice with normalized emittance of ∊_*x*_ = 0.5 nm rad. The novel feature of the MBA lattice was the use of seven vertically focusing bend magnets with different bending angles throughout the achromat cell to keep the radiation integrals and resulting beam emittance low. The baseline design called for a 400 m ring circumference with 12 straight sections of 6 m length. The dynamic aperture behaviour of the DLSR lattice was estimated to produce > 5 h beam lifetime at 100 mA stored beam current.

## Abstract 2

The first proposed lattice for a ‘diffraction-limited light source’ is reported. This approach has now more or less been used for the MAX IV project.

## 1. Introduction   

One of the first proposals for the lattice design of a diffraction-limited light source was produced by the authors in the early 1990s. At that time the new third-generation light sources ALS, ESRF and ELETTRA were commissioned in record times. All three machines reached their target specifications without any significant beam-dynamic problems and have operated reliably ever since. The successful commissioning results gave us confidence to expect that it was possible to operate storage rings with horizontal emittance an order of magnitude lower, in the 0.5 nm rad range. With a coupling of 1%, the vertical emittance would be of order 5 pm rad. For this special issue of the *Journal of Synchrotron Radiation*, we summarize the early design efforts in the 1990s and with hindsight relate those proposals to present storage-ring light-source lattice designs (Einfeld & Plesko, 1993*a* ▶,*b* ▶; Einfeld *et al.*, 1994*a* ▶, 1995 ▶). Having performed the original calculations over 20 years ago, we are thrilled with the prospect that the new MAX IV facility will be the first synchrotron radiation light source to implement a sub-nm rad variant of this design and that other proposed machines are following the MBA concept.

One of the most important factors for synchrotron radiation research is the photon beam brilliance which in storage rings is determined by the electron beam emittance and coupling between horizontal and vertical planes. Even in the limit of zero beam emittance, however, the phase space of the radiation emission from an undulator is itself finite due to diffraction effects at the source. For single-mode photon emission, the corresponding diffraction-limited ‘emittance’ of the photon beam is given by
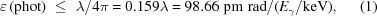
where λ is the X-ray wavelength and *E*_γ_ is the photon energy in keV (Coisson, 1988 ▶; Wiedemann, 2002 ▶). For photon energies of 1, 5 and 10 keV, the corresponding beam emittance should be smaller than 100, 20 and 10 pm rad, respectively. Formula (1) has been much debated and there is still not full consensus that the factor 4π in the denominator is numerically correct (Onuki & Elleaume, 2003 ▶). Nevertheless, a light source is referred to as ‘diffraction-limited’ when the electron beam emittance is less than that of the radiated photon beam at the desired X-ray wavelength.

By way of review, recall that the horizontal emittance of an electron storage ring beam is determined by a balance between two competing processes: quantum excitation of betatron oscillations from photon emission and longitudinal re-acceleration within the RF cavities. The basic formula to calculate storage ring emittance, assuming isomagnetic bend magnets and no insertion devices, is summarized as (Ropert, 1998 ▶)
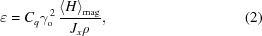
where *C*_*q*_ = 3.841 × 10^−13^ m is a constant, γ_o_ is the relativistic Lorentz factor, *J*_*x*_ is the Robinson partition number evaluated for the horizontal plane, ρ is the dipole magnet bending radius and 〈*H*〉_mag_ is the average of *H* evaluated in the bending magnets,
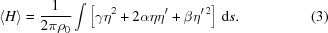
In (3), α, β and γ are the standard position-dependent Twiss parameters in the horizontal plane, and η, η′ are the horizontal dispersion function and first derivative, respectively. As a general rule for low emittance we aim to minimize the integral quantity 〈*H*〉_mag_ to maintain sufficient straight-section free space for insertion devices and operate at high enough beam energy to meet the spectral requirements of the user community.

## 2. Low-emittance lattice design   

Referring again to equation (2), the horizontal beam emittance is seen to scale with the square of the beam energy, linearly with 〈*H*〉_mag_, and is inversely proportional to the partition number *J*_*x*_. For bending magnets with a pure dipole field, *J*_*x*_ = 1. Since the *H*-function is determined by the Twiss parameters and dispersion in bending magnets, low emittance can be reached if the beam σ-matrix and η(*s*) have specifically controlled values within the bending magnets. By way of example we can analytically evaluate the horizontal emittance for two well known examples: the first bend magnet of a flat-field double-bend achromat (DBA) lattice (Chasman & Green, 1975 ▶) and the centre bend magnet of a flat-field triple-bend achromat (TBA) structure (Einfeld & Muelhaupt, 1980 ▶).

For the DBA example, the horizontal dispersion function is zero (achromat) at the entrance of the first bend magnet of length *L*. The emittance is minimized when β_*x*_ has, at the distance S* from the beginning of the magnet, a minimum value β_min_ such that the following relations hold,
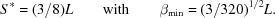
In this case, the emittance for the bending magnet of deflection angle ϕ is
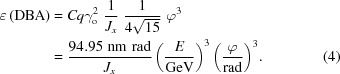
According to (4), the emittance scales with the well known third power law of deflection angle ϕ leading to the universal scaling law that a low-emittance lattice requires a large number of bending magnets.

For the second case we evaluate the centre bending magnet of a flat-field TBA lattice. Here the emittance is minimized when both β_*x*_ and η_*x*_ have minima in the middle of the centre bend at position (*L*/2). The corresponding theoretical minimum emittance (TME) conditions are (Sommer, 1983 ▶; Wüstefeld, 1987 ▶)
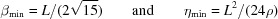
and the resulting TME emittance is
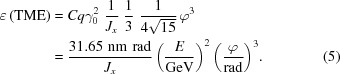
Closer inspection of (4) and (5) reveals that the TME emittance for the TBA magnet is a factor of three less than for the DBA magnet. Taking into account that the structure of a TBA must satisfy ‘Case 2’ in the centre and simultaneously satisfy achromatic ‘Case 1’ at the beginning and end bending magnets, the minimum emittance for a TBA lattice is, in practice,
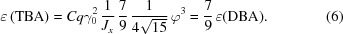
For the case when the bending-magnet deflection angle is the same for all three magnets, the advantage of the TBA structure over the DBA structure is only in the range of 23%. The reason for the discrepancy relative to the TBA-TME lattice is that the outer ‘achromat’ magnets dominate the emittance integral 〈*H*〉_mag_ producing an overall contribution of 85% to the emittance. Conversely, when we consider the case when the *total* deflection angle of the full TBA cell is the same as the DBA cell we have deflection angles ϕ(TBA) = (2/3)ϕ(DBA) and the emittance of the TBA cell is a factor of three less. An even smaller TBA emittance is achieved when the deflection angle of the outer magnets is further reduced and that of the middle magnet increased. For example, taking the bend angle of the outer magnets as half that of the centre dipole, the resulting cell (called TBA_mod_) has an emittance less than a quarter of the DBA cell,
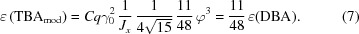
These examples demonstrate that in order to reach small emittance in an achromat cell the bending-magnet structure should be built up with bending magnets with different deflection angles: smaller magnets at the ends with larger angles in the centre. An application of this rule using a total of four bending magnets in a flat-field quadruple-bend achromatic (QBA) cell design is illustrated by Einfeld & Plesko (1993*b* ▶).

## 3. Low-emittance lattice with a modified QBA structure   

The most straightforward approach to QBA cell design involves directly inserting a second pair of bending magnets between the two bending magnets of a DBA structure with all magnets of the same deflection angle. The two dipoles in the centre of the cell should yield minimum emittance when the TME conditions leading up to equation (5) are satisfied. Similar to the DBA and TBA designs, the main contribution to emittance is given by the outer magnets in this case (2 × 37.5%). With the optimized TME settings mentioned above, the QBA design with four equal bend-magnet deflection angles yields ∊(QBA) = ∊(DBA)/12.

During the design phase for SPring-8, a comparison between DBA, TBA and QBA lattice cell structures was performed under the constraint that the ring circumference and the number of achromat cells was the same for all three cases (Tsumaki *et al.*, 1989*a* ▶,*b* ▶). Interestingly, all three lattices resulted in roughly the same beam emittance and any dependency of the emittance upon the deflection angle (∊ ≃ ϕ^3^) could not be found. The result of this study was to show that the standard QBA lattice described above has no advantage over standard DBA or TBA cell structures. As a result SPring-8 chose the DBA cell design to minimize construction risk.

**Figure 1:**
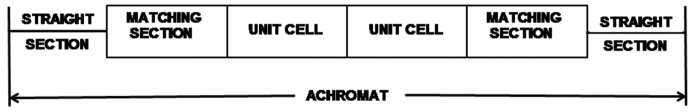
A modified QBA achromat’s inner structure. The deflection angle of the magnets in the unit cell is ϕ and in the matching section it is ϕ/2.

A quite different behaviour is found when the lattice design permits a modified QBA structure. We first proposed a modified QBA cell design during the planning phase for the synchrotron light source LISA (Einfeld *et al.*, 1992 ▶) with details described by Einfeld & Plesko (1993*b* ▶). As shown in Fig. 1 ▶, the modified QBA structure contains two internal ‘unit cells’ each with the deflection angle ϕ accompanied on each side by a matching section with deflection angle ϕ/2. Similar to lattice designs used for particle colliders, the matching sections force the dispersion to zero in the straight sections and permit optimum matching of the β-functions in the insertion-device straight sections. In order to achieve the desired Twiss parameters throughout the unit cells, focusing and a defocusing fields are needed. This was achieved in part by integrating vertical focusing into the dipoles (combined function magnets) along with standard standalone quadrupole magnets. The use of combined-function bending magnets increases the partition number *J*_*x*_ and yields an overall decrease in emittance of the QBA lattice by 30–40%. The novel introduction of internal unit cells composed of combined-function bending magnets provided the following advantages: (i) reduction in the total number of magnets per achromat; (ii) increase in horizontal partition number *J*_*x*_ (emittance reduction); (iii) creation of compact cells increasing straight-section space for insertion devices.

In terms of emittance, for the modified QBA lattice, ∊_*x*_ is again dominated by the bending magnets in the internal unit cells which gave an overall contribution of 2 × 42.1%. By switching from a standard QBA lattice to the modified QBA lattice the emittance is reduced by an additional factor of 2.53. The net result relative to the flat-field DBA lattice is **∊**(mod QBA) = **∊**(DBA)/30.36.

The extended concept of inserting multiple unit cells within two outer ‘matching’ cells at the ends of the achromat can be successfully generalized to obtain what can be called a multiple-bend achromat (MBA). An early example of a low-emittance MBA lattice was proposed for the 3 GeV synchrotron light source ROSY (Einfeld *et al.*, 1994*b* ▶). The ROSY design featured a small circumference of 148.1 m and relatively low emittance for such a small ring, 28.5 nm rad. In this case we designed the MBA cells with five bending magnets in an achromat configuration (5MBA). The deflection angle of the dipoles in the unit cells was 20° and in the matching sections 15°. As shown in the next section, the exercise of progressively reducing the bend angle of the individual dipole magnets while simultaneously increasing the number of unit cells within the achromat eventually leads to a low-emittance diffraction-limited light source.

## 4. Diffraction-limited light sources based on the MBA lattice   

**Figure 2:**
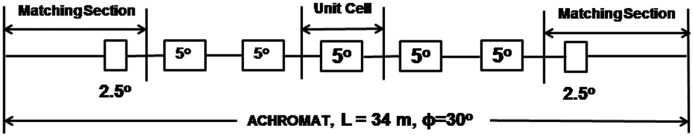
Layout for the diffraction-limited light source utilizing the MBA structure. The bending magnets are in the unit cell with a deflection of 5° and within the matching section with an angle of 2.5°. This is the magnetic structure which has been used for the lattice DIFL which is described by Einfeld & Plesko (1993*a* ▶), Einfeld *et al.* (1994*a* ▶) and Einfeld *et al.* (1995 ▶).

By fixing the electron beam energy at 3 GeV and scaling down the bend angle of the ROSY QBA cell to 5° per bend, we soon realised that it was possible to construct a diffraction-limited storage-ring light source with horizontal emittance ∊_*x*_ < 0.5 nm rad. Assuming a modest coupling factor of 3%, the vertical emittance is ∊_*y*_ = 12 pm rad. With five 5° unit cells per achromatic, the circumference of such a machine is approximately 400 m (see Fig. 2 ▶). The lattice for this layout has a 7MBA structure and was given the acronym ‘DIFL’. Although early results from the DIFL lattice investigation were published by Einfeld & Plesko (1993*a* ▶) and Einfeld *et al.* (1994*a* ▶, 1995 ▶), an outline of the design is repeated here to provide insight into the design process and to update the lattice calculations.

**Figure 3:**
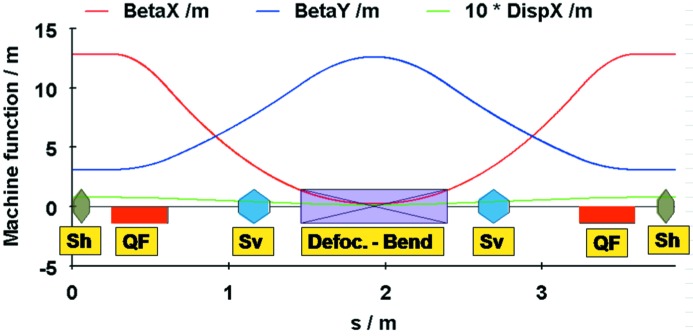
The arrangements of the magnets within a unit cell of the MBA and the corresponding machine functions. The parameters of the magnets are as follows. Bending: *L* = 0.931482 m, ρ = 10.674 m, *B* = 0.93749 T, *k* = −0.900 m^−2^. QF: *L* = 0.35 m, *k* = 1.992 m^−2^, *g* = 19.92 T m^−1^, *gL* = 6.972 T. Sh: *L* = 0.1 m, *m* = 53.347 m^−3^, ΣSh*L = 5.335 m^−2^. Sv: *L* = 0.2 m, m = −42.730 m^−3^, ΣSv*L = 8.546 m^−2^.

**Figure 4:**
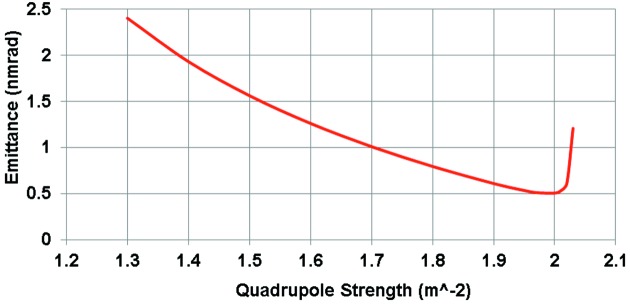
Emittance of the unit cell as a function of the strength of the focusing quadrupole.

**Figure 5:**
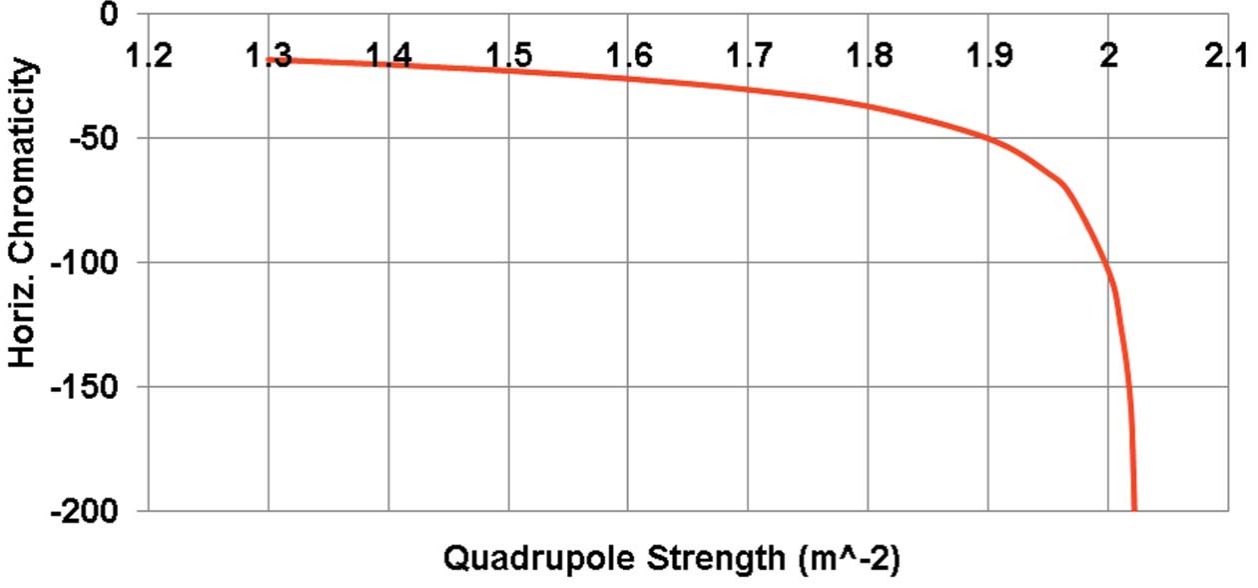
Horizontal chromaticity of the unit cell of the lattice DIFL.

As mentioned above, the emittance of an MBA achromatic cell is mainly determined by the bending angles of the internal unit cells. The DIFL lattice optimization was therefore first carried out using a hypothetical ring consisting of 72 5° unit cells (no matching cells). For purposes of this paper, all calculations have been performed with the lattice design code *OPA* (Streun, 2010 ▶). The layout of a single unit cell is presented in Fig. 3 ▶ with magnet field strengths listed in the caption. Unit-cell tuning is only possible by changing the strength of the QF magnet and field gradient in the bending magnet. The resulting beam emittance is plotted as a function of QF magnet strength in Fig. 4 ▶ and the horizontal chromaticity in Fig. 5 ▶. As seen from Fig. 4 ▶, the emittance goes through a minimum near *K*_QF_ = 2 m^−2^ and then increases rapidly. Similarly the natural horizontal chromaticity increases rapidly at higher values of *K*_QF_. Accordingly we set QF to achieve a minimum emittance value of 0.5 nm rad. Compared with the TME expression shown in equation (5), for a TME machine operating at 3 GeV with 5° bend magnets and *J*_*x*_ = 1.29, the minimum emittance is 0.15 nm rad. The 7MBA unit-cell design therefore has a higher emittance by a factor of only 3.3.

**Figure 6:**
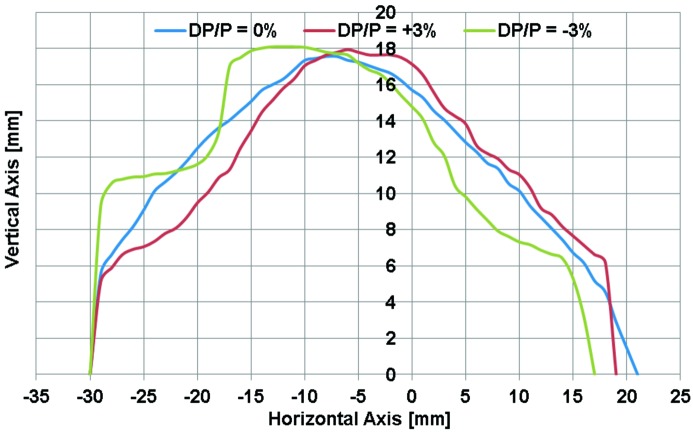
Dynamic aperture of the unit cell for the energy deviations Δ*p*/*p* = −3% (green line), Δ*p*/*p* = 0% (blue line), Δ*p*/*p* = 3% (red line).

To optimize the dynamic aperture the bending-magnet field gradient was set to maximize the ratio of β_*x*_ and β_*y*_ at the chromatic sextupole locations. The resulting dynamic aperture of a single unit cell is shown in Fig. 6 ▶ with both on-energy and off-energy beam conditions, δ*p*/*p* = 0% and ±3%. The bare-lattice dynamic aperture is in the range *x* = −30–20 mm and *y* = 14–16 mm, or, in terms of acceptance, *A*_*x*_ = 31π mm mrad and *A*_*y*_ = 83π mm mrad. This result is particularly large when evaluated relative to beam size: −300 to 180 times in the horizontal plane and ±2400 times in the vertical dimension.

Surprisingly, tracking studies showed the tune shift with the momentum with up to δ*p*/*p* up to ±3% changes by only 0.025. With a peak dispersion value of 0.075 m, an energy acceptance specification of 3% and a ratio of 2 for the β-functions from the arcs to the straights, an aperture of only ±3.2 mm is needed to capture Touschek-scattered particles. Hence the dynamic aperture should be sufficient for both injection and to yield an electron beam lifetime of the order of hours. Based on the simple unit-cell concept, in principle there were no ‘showstoppers’ to go forward with the 7MBA DIFL lattice design.

**Figure 7:**
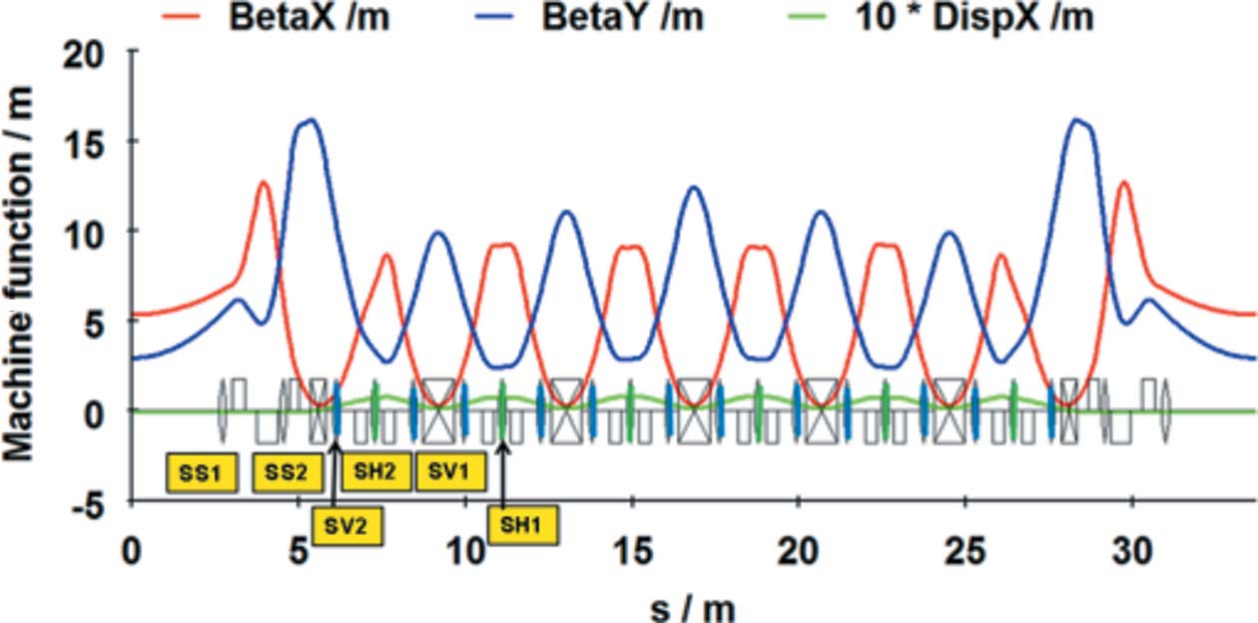
Machine function of the chosen lattice DIFL for the proposed diffraction-limited light source.

The Twiss parameters within a full achromat of the DIFL lattice including matching cells are plotted in Fig. 7 ▶. In order to optimized the dynamic aperture, tune shift with amplitude and tune shift with momentum deviation, additional sextupole families SS1 and SS2 were introduced. Sextupole families SV1 and SH1 have the same settings for all unit cells. The main characteristics of the ∊_*x*_ = 0.5 nm rad DIFL lattice are given in Table 1 ▶. As expected, the emittance remains at ∊_*x*_ ≃ 0.5 nm rad even with the addition of the outer matching cell dipole magnets. Assuming 1% coupling, the r.m.s. beam size is σ_*x*_ = 52 µm and σ_*y*_ = 3.8 µm in the straight sections.

**Figure 8:**
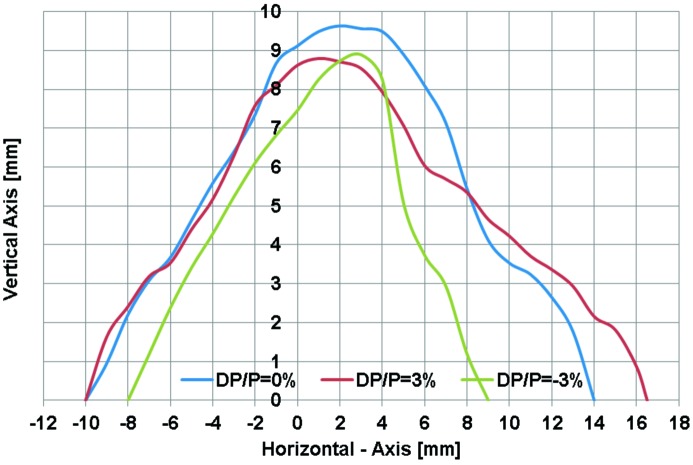
Dynamic aperture of the lattice DIFL for the energy deviations Δ*p*/*p* = −3% (red line), Δ*p*/*p* = 0% (black line), Δ*p*/*p* = 3% (blue line).

**Figure 9:**
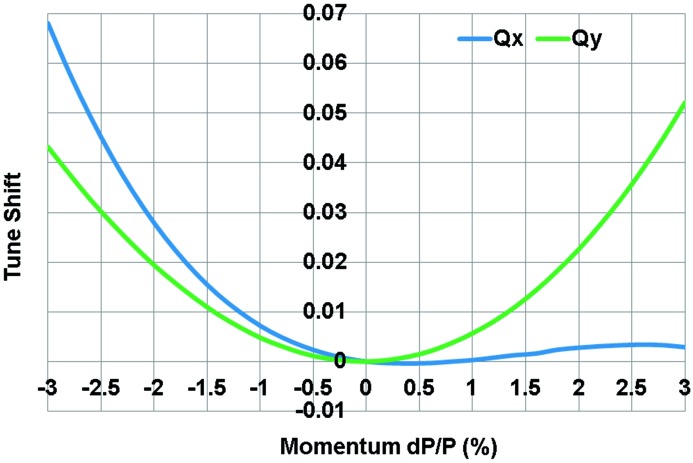
Change of the tunes for energy deviations of up to ±3%.

The dynamic aperture of the DIFL lattice is plotted in Fig. 8 ▶, along with the tune shift as a function of momentum deviation in Figs. 9 ▶ and 10 ▶. For energy deviations δ*p*/*p* = ±3%, the horizontal dynamic aperture remains in the range ±10 mm and in the vertical ±8 mm. In terms of acceptance, we have *A*_*x*_ = 17.7π mm mrad and *A*_*y*_ = 21.7π mm mrad. Comparing with the lattice design composed of unit cells only, the dynamic acceptance is reduced by factors of 1.75 and 3.82 in the horizontal and vertical planes, respectively.

To complete the conceptual design we assumed a standard 500 MHz RF acceleration system with a factor of three overvoltage and 1% coupling and calculate an electron beam lifetime of ∼5 h. The electron beam energy spread is 0.85 × 10^−3^, the natural chromaticities are manageable (ξ_*x*_ = −79.4ξ_*y*_ = −38.5) and the damping times are of order 10 ms. The betatron tunes are *Q*_*x*_ = 37.82 and *Q*_*y*_ = 13.20, well away from dangerous resonances.

**Figure 11:**
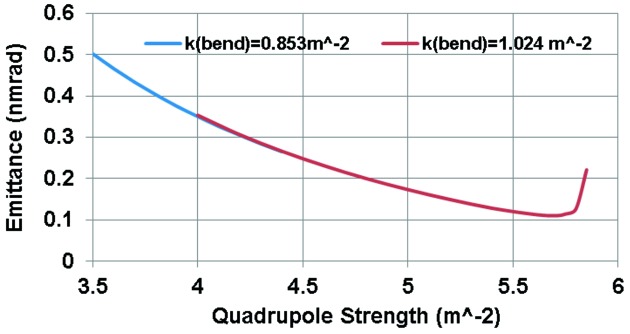
Emittance of the unit cell of MAX IV as a function of the strength of the focusing quadrupole.

As a final example we now examine the lattice design for MAX IV (Tavares *et al.*, 2014 ▶). MAX IV has roughly the same magnet structure as the 7MBA DIFL with the unit-cell dipole bending angles reduced to 3° and the matching cell dipoles 1.5°. The unit-cell emittance is plotted as a function of QF strength in Fig. 11 ▶. From this plot it is clear that the minimum emittance for the case with QF = 5.7 m^−2^ is around 0.12 nm rad. Referring again to the expression for TME [equation (5)], the emittance of a 3 GeV TME storage ring with a dipole deflection angles of 3° and *J*_*x*_ = 2.07 is 0.02 nm rad, or a factor of six below the MAX IV value.

Detailed tracking studies of MAX IV show that the dynamic aperture is too small with a beam emittance of 0.12 nm rad so the QF strength was reduced to 4 m^−2^ yielding ∊_*x*_ = 0.33 nm rad for the unit cell (factor of 16.5 below the TME value). The bore radius of the MAX IV magnets is 12.5 mm (Johansson *et al.*, 2014 ▶) producing pole tip fields of 0.5 T in the quadrupoles and 0.33 T in the 207 m^−3^ sextupole magnets. The integrated strengths of the dipole, quadrupole and sextupole magnets are roughly the same as the unit cell of the DIFL. Given the small magnet radii at MAX IV, the field strength can be much higher which leads to a compact lattice. In this case, the overall length of each cell is reduced by 7.3 m. The resulting ring circumference with 12 achromat cells is therefore reduced by 84 m relative to the DIFL design. For the final MAX IV machine configuration, 20 achromat cells were used to produce an overall ring circumference of 528 m and horizontal emittance ∊_*x*_ = 0.33 nm rad.

## 5. Conclusions   

It is well known that the emittance of a storage ring synchrotron light source scales with the third power of the bending-magnet deflection angles. In order to keep the accelerator compact and overall size reasonable, we have systematically demonstrated how a lattice with many bending magnets integrated into each achromat cell leads to an optimal design. In general, the MBA lattice provides a means to obtain lower emittance and higher dynamic apertures than with classical DBA lattices. The advantages of the combined-function MBA lattice include the small contribution of the outer bending magnets to emittance, a large horizontal partition number, a low number of quadrupole magnets, short unit-cell lengths and relatively simple chromatic correction. The historical investigation of the seven-dipole MBA revealed that the emittance of a diffraction-limited light source with values below 0.5 nm rad was possible. Further advances are of course possible by permitting finite dispersion in the straight sections and with the introduction of sophisticated sextupole magnet arrangements. Nevertheless, the original design proposed over 20 years ago has proved its validity and opened up new R&D paths that are fully blossoming today.

## Figures and Tables

**Figure 10:**
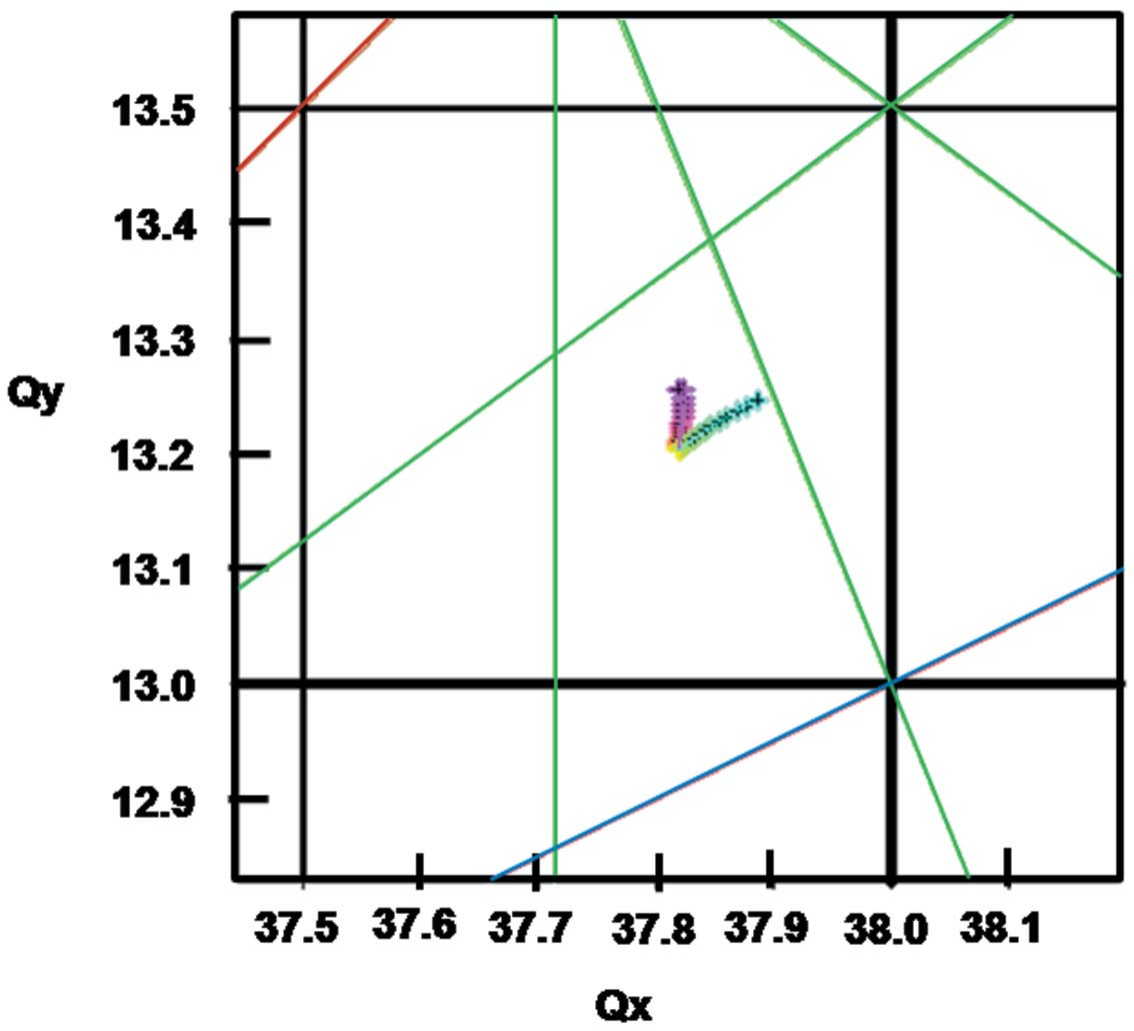
Tune diagram for the lattice DIFL with the movement of the working point for energy deviations up to ±3%. The resonance lines are the following: blue = third order, brown = fourth order, green = seventh order.

**Table 1:** Main characteristics of the DIFL lattice

Circumference (m)	*C*	404.3
Number of achromats		12
Energy (GeV)	*E*	3
Natural emittance (nm rad)	∊	0.5
Coupling factor (%)		1
Horizontal tune	*Q*_*x*_	37.82
Vertical tune	*Q*_*y*_	13.204
Horizontal chromaticity	ξ_*x*_	−79.4
Vertical Chromaticity	ξ_*y*_	−38.5
Momentum compaction factor	α	0.237 × 10^−4^
Natural energy spread	σ_*E*_/*E*	0.852 × 10^−3^
Partition number	*J*_*x*_	1.32
Energy loss per turn (keV)	*U*_0_	672
Horizontal β-function	β_*x*_(0)	5.345
Vertical β-function	β_*y*_(0)	2.944
Horizontal beam size (µm)	σ_*x*_(0)	52.3
Vertical beam size (µm)	σ_*y*_(0)	3.8
